# A Unified Classification of Alien Species Based on the Magnitude of their Environmental Impacts

**DOI:** 10.1371/journal.pbio.1001850

**Published:** 2014-05-06

**Authors:** Tim M. Blackburn, Franz Essl, Thomas Evans, Philip E. Hulme, Jonathan M. Jeschke, Ingolf Kühn, Sabrina Kumschick, Zuzana Marková, Agata Mrugała, Wolfgang Nentwig, Jan Pergl, Petr Pyšek, Wolfgang Rabitsch, Anthony Ricciardi, David M. Richardson, Agnieszka Sendek, Montserrat Vilà, John R. U. Wilson, Marten Winter, Piero Genovesi, Sven Bacher

**Affiliations:** 1 Institute of Zoology, Zoological Society of London, London, United Kingdom; 2 Distinguished Scientist Fellowship Program, King Saud University, Riyadh, Saudi Arabia; 3 Environment Institute, School of Earth & Environmental Sciences, University of Adelaide, Adelaide, South Australia, Australia; 4 Department of Conservation Biology, Vegetation and Landscape Ecology, University of Vienna, Vienna, Austria; 5 Imperial College London, Ascot, Berkshire, United Kingdom; 6 The Bio-Protection Research Centre, Lincoln University, Christchurch, New Zealand; 7 Technische Universität München, Department of Ecology and Ecosystem Management, Restoration Ecology, Freising-Weihenstephan, Germany; 8 UFZ - Helmholtz Centre for Environmental Research, Department of Community Ecology, Halle, Germany; 9 German Centre for Integrative Biodiversity Research (iDiv) Halle-Jena-Leipzig, Leipzig, Germany; 10 Centre for Invasion Biology, Department of Botany and Zoology, Stellenbosch University, Matieland, South Africa; 11 Institute of Botany, Department of Invasion Ecology, Academy of Sciences of the Czech Republic, Průhonice, Czech Republic; 12 Department of Ecology, Faculty of Science, Charles University in Prague, Prague, Czech Republic; 13 Institute of Ecology and Evolution, University of Bern, Bern, Switzerland; 14 Environment Agency Austria, Department of Biodiversity and Nature Conservation, Vienna, Austria; 15 Redpath Museum, McGill University, Montreal, Quebec, Canada; 16 Estación Biológica de Doñana (EBD-CSIC), Seville, Spain; 17 South African National Biodiversity Institute, Kirstenbosch National Botanical Gardens, Claremont, South Africa; 18 ISPRA, Institute for Environmental Protection and Research and Chair IUCN SSC Invasive Species Specialist Group, Rome, Italy; 19 Department of Biology, Unit Ecology & Evolution, University of Fribourg, Fribourg, Switzerland

## Abstract

Species moved by human activities beyond the limits of their native geographic ranges into areas in which they do not naturally occur (termed aliens) can cause a broad range of significant changes to recipient ecosystems; however, their impacts vary greatly across species and the ecosystems into which they are introduced. There is therefore a critical need for a standardised method to evaluate, compare, and eventually predict the magnitudes of these different impacts. Here, we propose a straightforward system for classifying alien species according to the magnitude of their environmental impacts, based on the mechanisms of impact used to code species in the International Union for Conservation of Nature (IUCN) Global Invasive Species Database, which are presented here for the first time. The classification system uses five semi-quantitative scenarios describing impacts under each mechanism to assign species to different levels of impact—ranging from Minimal to Massive—with assignment corresponding to the highest level of deleterious impact associated with any of the mechanisms. The scheme also includes categories for species that are Not Evaluated, have No Alien Population, or are Data Deficient, and a method for assigning uncertainty to all the classifications. We show how this classification system is applicable at different levels of ecological complexity and different spatial and temporal scales, and embraces existing impact metrics. In fact, the scheme is analogous to the already widely adopted and accepted Red List approach to categorising extinction risk, and so could conceivably be readily integrated with existing practices and policies in many regions.

## Abstract 2

We present a method for categorising and comparing alien or invasive species in terms of how damaging they are to the environment, that can be applied across all taxa, scales, and impact metrics.

## Introduction

Human activities are transforming natural environments by moving species beyond the limits of their native geographic ranges into areas in which they do not naturally occur. Many of these alien species (Box 1) have caused substantial changes to the recipient ecosystems. Such changes have been measured by a burgeoning number of studies that consider a broad range of environmental impacts, defined here as measurable changes to the properties of an ecosystem by an alien species [1],[2], at different levels of organisation (Box 1). For example, alien species have been shown to cause significant changes in native species extinction probabilities, genetic composition of native populations, behaviour patterns, species richness and abundance, phylogenetic and taxonomic diversity, trophic networks, ecosystem productivity, nutrient and contaminant cycling, hydrology, habitat structure, and various components of disturbance regimes [1]–[8]. Such changes are often indirect, and may involve subtle or poorly studied interactions that could yield substantial effects over time [9]. For these reasons, most scientists and conservation organisations consider alien species to be undesirable additions to ecosystems, and frequently devote considerable resources towards preventing or mitigating their impacts.

**Box 1. A Glossary of Key Definitions**

**Alien species**: a species moved by human activities beyond the limits of its native geographic range into an area in which it does not naturally occur. The movement allows the species to overcome fundamental biogeographic barriers to its natural dispersal. Common synonyms are exotic, introduced, non-indigenous, or non-native [50].

**Environmental impact**: a measurable change to the properties of an ecosystem by an alien species [2]. Our definition means that our scheme applies to all ecosystems—whether largely natural or largely managed by humans—but explicitly considers only effects that have impacts on the native biota or the ecosystem processes that derive from that environment. The same alien species may also have impacts on human societies and economies [37], but these represent additional and complex dimensions of impacts [51]–[56], and one should avoid conflating environmental with non-environmental impacts.

**Deleterious impact**: an impact that changes the environment in such a way as to reduce native biodiversity or alter ecosystem function to the detriment of the incumbent native species—as indicated by a change in importance or abundance following invasion. This is similar to the “adverse effect” concept [57]. This definition intentionally excludes societal judgments regarding the desirability or value of aliens, although our assumption is that the classification will be used as a mechanism to prevent impacts that are judged to be “negative” by those concerned.

**Figure 1:**
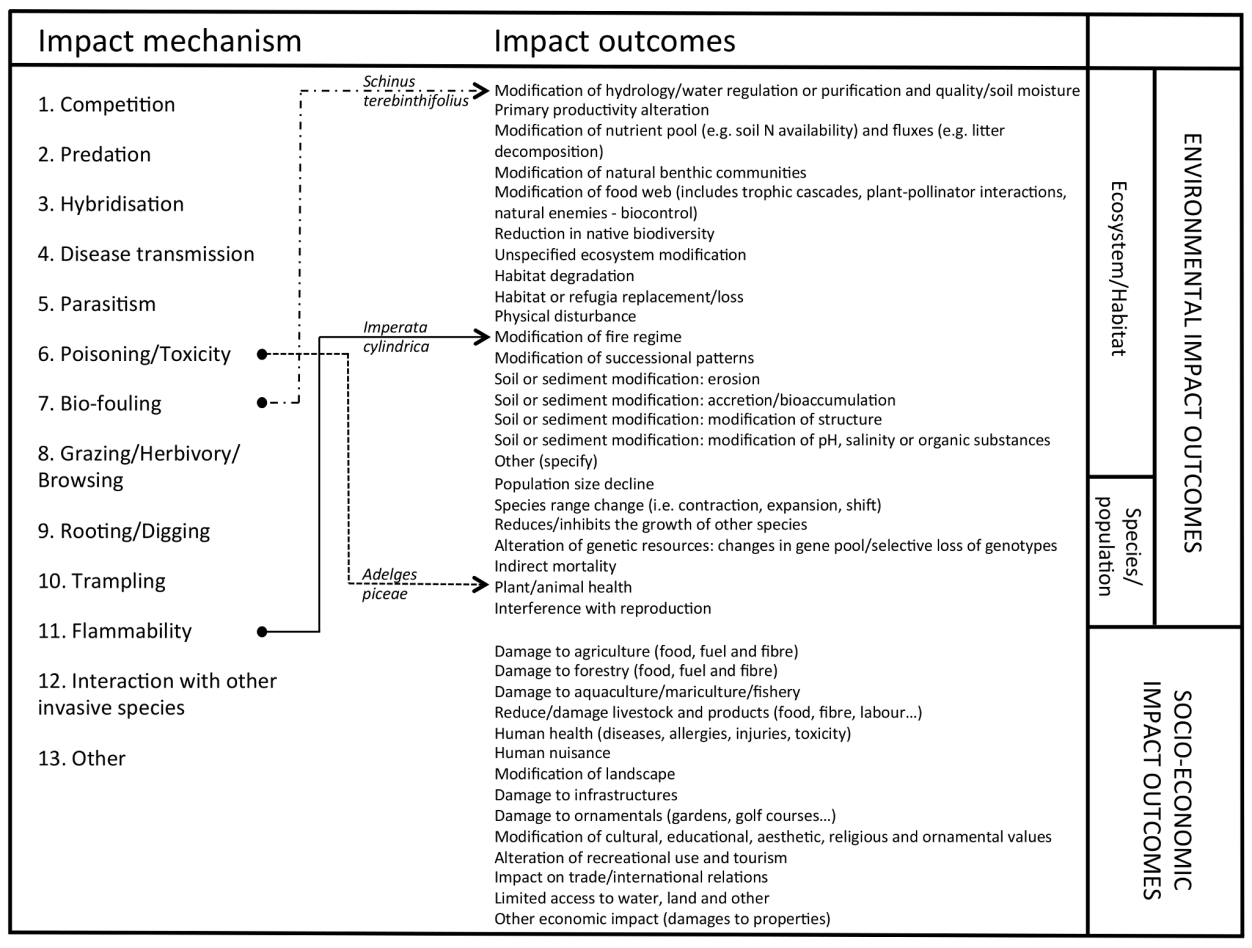
Impact scheme of the Global Invasive Species Database, implemented by the IUCN Species Survival Commission (SSC) Invasive Species Specialist Group. The GISD stores detailed information on more than 800 invasive alien species, including on the impacts they cause. The GISD has recently been redesigned, and all information has been re-classified in order to improve the searching functionalities of the database. The schema developed for the revised GISD has allowed all species stored in the database to be coded in respect of the direct mechanisms by which their impacts occur (e.g., predation), and by the outcomes of those impact mechanisms on the environment or on human activities. For example, the grass *Imperata cylindrica* (Poales: Poaceae) almost doubles litter biomass in invaded locations, which increases potential fuel for fires (impact mechanism coded as flammability, and impact outcome as modification of fire regime). The plant *Schinus terebinthifolius* (Sapindales: Anacardiaceae) is a bio-fouling agent, forming dense thickets in gullies and river bottoms, with the ultimate effect of changing the hydrology of river streams of invaded freshwater bodies (mechanism coded as bio-fouling, and impact outcome described as modification of hydrology). The insect *Adelges piceae* (Hemiptera: Adelgidae) releases a toxin causing stress to trees, which eventually die. The impact outcome of *A. piceae* is described in GISD as damage to forestry, with its mechanism of impact coded as poisoning/toxicity, but it can also be coded as having an environmental impact on plant/animal health, as it has been here. In the table, mechanisms and outcomes are reported in two separate columns, and the three examples of the connections between mechanisms and outcomes are shown. Impact outcomes in the GISD database can be environmental or socio-economic, but our categorisation scheme of species in terms of the magnitudes of their impacts (Figure 2; Table 1) concerns only the former.

**Impact mechanisms**: categories into which different types of alien species impact are classified. The IUCN GISD identifies 13 such categories; a list of these impact mechanisms is given in Figure 1.

**Propagule pressure**: a composite measure of the number of individuals that are released or escape into a region to which they are not native. It incorporates estimates of the absolute number of individuals involved in any one release/escape event (propagule size) and the number of discrete such events (propagule number) [58].

**Residence time**: the length of time that an alien species has been in its introduced range [59].

However, many alien species apparently have had little or no detectable effects on their new environment [1],[10],[11], and some effects may be considered to be positive [12]–[16]. It has been further claimed that alien species are no more likely to have undesirable impacts than natives, and therefore that management attention should be based on impacting species in general, rather than on the alien/native origin of species [17],[18], although this view is controversial [19],[20]. These commentators urge conservationists and land managers to organise priorities around whether species are producing net benefits or harm, so as to avoid wasting valuable conservation resources on the costs of excluding (e.g., through ballast-water treatment), eradicating, containing, or controlling alien species [21]. Recognising that impacts vary greatly among species and among recipient systems, and that many notable impacts only become obvious or significantly influential long after the onset of invasion, a critical need for invasion biology is the capacity to evaluate, compare, and predict the magnitudes of the impacts of different alien species, in order to determine and prioritise appropriate actions where necessary. The challenge is how to compare impacts attributable to diverse alien taxa on different levels of ecological complexity (individuals, populations, communities, ecosystems), at different spatial and temporal scales, assessed using a range of metrics and techniques [22].

In response to these issues, here we propose a simple standardised system for classifying alien species in terms of the magnitude of their impacts. Our aim is to produce a practical tool to report on the impacts caused by alien species, that can (i) be used to identify those species that have different levels of environmental impact, (ii) facilitate comparisons of the level of impact from alien species among regions and taxa, (iii) facilitate predictions of potential future impacts of the species in the target region and elsewhere, (iv) align with the mechanisms of impact identified in the International Union for Conservation of Nature (IUCN) Global Invasive Species Database ([GISD]; http://www.issg.org/database), and (v) prioritise management actions. The system we propose has the following properties, many of which also underlie the intentions of the IUCN Red List categories and criteria (our classification system can be viewed as broadly analogous to that approach) [23]:

1. The classification considers only environmental (as opposed to economic or societal) impacts (see Box 1 for definitions). Nevertheless, our scheme could be extended to social and economic impacts, as well as to environmental impacts on resident alien species that are perceived to be harmless or beneficial.
2. The classification identifies species that have deleterious abiotic or biotic impacts (Box 1). Its aim is not to weigh deleterious against beneficial impacts to determine the net value of an introduction, but rather to highlight potential consequences.
3. Species are classified on the basis of evidence of their most severe documented impacts in regions to which they have been introduced. The scheme is, therefore, not a predictive model of impact—however, by reporting on the worst observed case, it can be used to flag species with high potential impacts that need to be evaluated in detail in a particular introduction context.
4. The classification provides a consistent procedure for translating the broad range of impact types and measures into ranked levels of environmental impact. It therefore distinguishes between taxa with different magnitudes of impact.
5. The classification can be applied across taxa, so that different taxa can be compared using a common currency in terms of their environmental impact. It could also be applied at different taxonomic levels.
6. The classification considers consequences, not likelihoods; that is, it focuses on the consequences in terms of impact of an introduction, rather than on the likelihood of an invasion.
7. Classification is based on the best available evidence. Hence, species can move up and down impact categories as the quality of evidence improves, as conditions change, or as an invasion proceeds.
8. The scheme we propose here can be applied to impacts assessed at a range of spatial scales, from global to national or regional.

## Classifying Impact

Our classification system is based on the Generic Impact Scoring System (GISS) to compare the impacts of alien animal species among members of large taxonomic groups, developed by Nentwig and colleagues [24] and subsequently extended by Kumschick and colleagues [25], modified to align it to the new impact scheme of the GISD implemented by the IUCN Species Survival Commission (SSC) Invasive Species Specialist Group (Figure 1).

The extended GISS [25] identified a set of six impact classes (herbivory; competition; predation; disease transmission; hybridisation; impact on ecosystem, other than those mentioned before, i.e., chemical, physical, or structural changes), which we here term impact mechanisms (Box 1). Each of these mechanisms was associated with one of a sequential series of six impact scenarios (ranked 0–5) describing increasing levels of impact by aliens by that mechanism. These semi-quantitative scenarios were designed such that each step change in category reflects an increase in the order of magnitude of the particular impact so that a new level of organisation is involved. Thus: (0) no discernible impact; (1) discernible impacts, but no effects on individual fitness; (2) effects on fitness, but not on populations; (3) changes to populations, but not to community composition; (4) community changes, which are reversible; and (5) irreversible community changes and extinctions. Species impacts are assessed and assigned to a scenario for each impact mechanism. The scenario ranks assigned for each impact mechanism can be summed to produce an overall impact score. Species can then be compared with respect to these scores, for example to identify traits associated with higher levels of impact [24],[26].

Our classification scheme is based on the impact mechanisms and scenarios presented by Kumschick and colleagues [25], but modified in four ways. First, and most importantly, we added new scenarios for mechanisms of impact additionally identified in the IUCN GISD. The GISD scheme identifies 13 impact mechanisms (Figure 1), along with associated outcomes of those impacts in respect of changes to environmental or socio-economic parameters. Of these, numbers 1–4 and 8 correspond directly to scenarios in Kumschick and colleagues [25], while mechanisms 9–11 are captured under Kumschick and colleague's mechanism of impact on ecosystem (see above). We therefore expanded the Kumschick and colleagues scheme by adding explicit scenarios for four mechanisms of impact in the GISD schema: parasitism (impact 5 in Figure 1), poisoning/toxicity (impact 6), bio-fouling (impact 7), and interaction with other invasive species (impact 12). We ignored the thirteenth mechanism (other), as it is not possible to derive scenarios for unspecific impacts (although impacts not captured by the first 12 mechanisms can still be assigned on the basis of the general meanings identified in Table 1).

**Table 1:** Impact criteria for assigning alien species to different categories in the classification scheme (Box 2).

Impact Class	Massive (MA)	Major (MR)	Moderate (MO)	Minor (MI)	Minimal (ML)
***Categories should adhere to the following general meaning***	*Causes at least local extinction of species, and irreversible changes in community composition; even if the alien species is removed the system does not recover its original state*	*Causes changes in community composition, which are reversible if the alien species is removed*	*Causes declines in population densities, but no changes in community composition*	*Causes reductions in individual fitness, but no declines in native population densities*	*No effect on fitness of individuals of native species*
**Competition (1)**	Competition resulting in replacement or local extinction of one or several native species; changes in community composition are irreversible	Competition resulting in local or population extinction of at least one native species, leading to changes in community composition, but changes are reversible when the alien species is removed	Competition resulting in a decline of population size of at least one native species, but no changes in community composition	Competition affects fitness (e.g., growth, reproduction, defence, immunocompetence) of native individuals without decline of their populations	Negligible level of competition with native species; reduction of fitness of native individuals is not detectable
**Predation (2)**	Predators directly or indirectly (e.g., via mesopredator release) resulting in replacement or local extinction of one or several native species (i.e., species vanish from communities at sites where they occurred before the alien arrived); changes in community composition are irreversible	Predators directly or indirectly (e.g., via mesopredator release) resulting in local or population extinction of at least one native species, leading to changes in community composition, but changes are reversible when the alien species is removed	Predators directly or indirectly (e.g., via mesopredator release) resulting in a decline of population size of at least one native species but no changes in community composition	Predators directly or indirectly (e.g., via mesopredator release) affecting fitness (e.g., growth, reproduction) of native individuals without decline of their populations	Negligible level of predation on native species
**Hybridisation (3)**	Hybridisation between the alien species and native species is common in the wild; hybrids are fully vigorous and fertile; pure native species cannot be recovered by removing the alien, resulting in replacement or local extinction of native species by introgressive hybridisation (genomic extinction)	Hybridisation between alien species and native species is common in the wild; F1 hybrids are vigorous and fertile, however offspring of F1 hybrids are weak and sterile (hybrid breakdown), thus limited gene flow between alien and natives; individuals of alien species and hybrids discernible from pure natives, pure native populations can be recovered by removing the alien and hybrids.	Hybridisation between alien species and native species is regularly observed in the wild; hybrids are vigorous, but sterile (reduced hybrid fertility),limited gene flow between alien and natives, local decline of populations of pure native species, but pure native species persists	Hybridisation between alien species and native species is observed in the wild, but rare; hybrids are weak and never reach maturity (reduced hybrid viability), no decline of pure native populations	No hybridisation between alien species and native species observed in the wild (prezygotic barriers), hybridisation with a native species might be possible in captivity
**Transmission of diseases to native species (4)**	Transmission of diseases to native species resulting in replacement or local extinction of native species (i.e., species vanish from communities at sites where they occurred before the alien arrived); changes in community composition are irreversible	Transmission of diseases to native species resulting in local or population extinction of at least one native species, leading to changes in community composition, but changes are reversible when the alien species is removed	Transmission of diseases to native species resulting in a decline of population size of at least one native species, but no changes in community composition	Transmission of diseases to native species affects fitness (e.g., growth, reproduction, defence, immunocompetence) of native individuals without decline of their populations	The alien species is not a host of diseases transmissible to native species or very low level of transmission of diseases to native species; reduction of fitness of native individuals is not detectable
**Parasitism (5)**	Parasites or pathogens directly or indirectly (e.g., apparent competition) resulting in replacement or local extinction of one or several native species (i.e., species vanish from communities at sites where they occurred before the alien arrived); changes in community composition are irreversible	Parasites or pathogens directly or indirectly (e.g., apparent competition) resulting in local or population extinction of at least one native species, leading to changes in community composition, but changes are reversible when the alien species is removed	Parasites or pathogens directly or indirectly (e.g., apparent competition) resulting in a decline of population size of at least one native species but no changes in community composition	Parasites or pathogens directly or indirectly (e.g., apparent competition) affecting fitness (e.g., growth, reproduction, defence, immunocompetence) of native individuals without decline of their populations	Negligible level of parasitism or disease incidence (pathogens) on native species, reduction of fitness of native individuals is not detectable
**Poisoning/toxicity (6)**	The alien species is toxic/allergenic by ingestion, inhalation, or contact to wildlife or allelopathic to plants, resulting in replacement or local extinction of native species; changes in community composition are irreversible	The alien species is toxic/allergenic by ingestion, inhalation, or contact to wildlife or allelopathic to plants, resulting in local or population extinction of at least one native species (i.e., species vanish from communities at sites where they occurred before the alien arrived), leading to changes in community composition, but changes are reversible when the alien species is removed	The alien species is toxic/allergenic by ingestion, inhalation, or contact to wildlife or allelopathic to plants, resulting in a decline of population size of at least one native species, but no changes in community composition (native species richness)	The alien species is toxic/allergenic by ingestion, inhalation, or contact to wildlife or allelopathic to plants, affects fitness (e.g., growth, reproduction, defence, immunocompetence) of native individuals without decline of their populations	The alien species is not toxic/allergenic/allelopathic, or if it is, the level is very low, reduction of fitness of native individuals is not detectable
**Bio-fouling (7)**	Bio-fouling resulting in replacement or local extinction of one or several native species (i.e., species vanish from communities at sites where they occurred before the alien arrived); changes in community composition are irreversible	Bio-fouling resulting in local or population extinction of at least one native species, leading to changes in community composition, but changes are reversible when the alien species is removed	Bio-fouling resulting in a decline of population size of at least one native species, but no changes in community composition	Bio-fouling affects fitness (e.g., growth, reproduction, defence, immunocompetence) of native individuals without decline of their populations	Negligible level of bio-fouling on native species; reduction of fitness of native individuals is not detectable
**Grazing/herbivory/browsing (8)**	Herbivory resulting in replacement or local extinction of one or several native plant species (i.e., species vanish from communities at sites where they occurred before the alien arrived); changes in community composition are irreversible	Herbivory resulting in local or population extinction of at least one native plant species, leading to changes in community composition, but changes are reversible when the alien species is removed	Herbivory resulting in a decline of population size of at least one native species, but no changes in community composition	Herbivory affects fitness (e.g., growth, reproduction, defence, immunocompetence) of individual native plants without decline of their populations	Negligible level of herbivory on native plant species, reduction of fitness on native plants is not detectable
**Chemical, physical, or structural impact on ecosystem (9, 10, 11)**	Many changes in chemical, physical, and/or structural biotope characteristics; or changes in nutrient and water cycling; or disturbance regimes; or changes in natural succession, resulting in replacement or local extinction of native species (i.e., species vanish from communities at sites where they occurred before the alien arrived); changes (abiotic and biotic) are irreversible	Changes in chemical, physical, and/or structural biotope characteristics; or changes in nutrient cycling; or disturbance regimes; or changes in natural succession, resulting in local extinction of at least one native species, leading to changes in community composition, but changes are reversible when the alien species is removed	Changes in chemical, physical, and/or structural biotope characteristics; or changes in nutrient cycling; or disturbance regimes; or changes in natural succession, resulting in a decline of population size of at least one native species, but no changes in community composition	Changes in chemical, physical, and/or structural biotope characteristics; or changes in nutrient cycling; or disturbance regimes; or changes in natural succession detectable, affecting fitness (e.g., growth, reproduction, defence, immunocompetence) of native individuals without decline of their populations	No changes in chemical, physical, and/or structural biotope characteristics; or changes in nutrient cycling; or disturbance regimes; or changes in natural succession detectable, or changes are small with no reduction of fitness of native individuals detectable
**Interaction with other alien species (12)**	Interaction of an alien species with other aliens (e.g., pollination, seed dispersal, habitat modification) facilitates replacement or local extinction of one or several native species (i.e., species vanish from communities at sites where they occurred before the alien arrived), and produces irreversible changes in community composition that would not have occurred in the absence of the species. These interactions may be included in other impact classes (e.g., predation, apparent competition) but would not have resulted in the particular level of impact without an interaction with other alien species	Interaction of an alien species with other aliens (e.g., pollination, seed dispersal, habitat modification) facilitates local or population extinction of at least one native species, and produces changes in community composition that are reversible but would not have occurred in the absence of the species. These interactions may be included in other impact classes (e.g., predation, apparent competition) but would not have resulted in the particular level of impact without an interaction with other alien species	Interaction of an alien species with other aliens (e.g., pollination, seed dispersal, habitat modification) facilitates a decline of population size of at least one native species, but no changes in community composition; changes would not have occurred in the absence of the species. These interactions may be included in other impact classes (e.g., predation, apparent competition) but would not have resulted in the particular level of impact without an interaction with other alien species	Interaction of an alien species with other aliens (e.g., pollination, seed dispersal) affects fitness (e.g., growth, reproduction, defence, immunocompetence) of native species' individuals without decline of their populations; changes would not have occurred in the absence of the species. These interactions may be included in other impact classes (e.g., predation, apparent competition) but would not have resulted in the particular level of impact without an interaction with other alien species	Interaction of an alien species with other aliens (e.g., pollination, seed dispersal) but with minimal effects on native species; reduction of fitness of native individuals is not detectable

**Figure 2:**
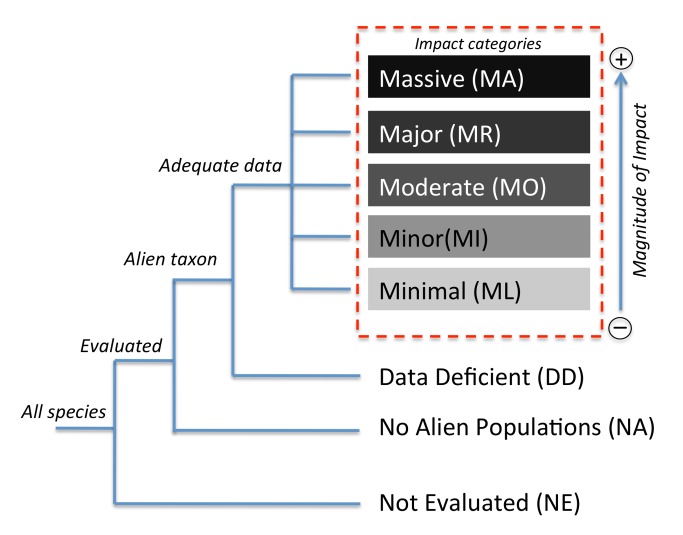
The different categories in the alien species impact scheme, and the relationship between them. Descriptions of the categories are provided in Box 2. The CG category is not represented in this diagram as CG taxa may be found in any category.

These categories are for species that have been evaluated, have alien populations (i.e., are known to have been introduced outside their native range), and for which there is adequate data to allow classification (see Figure 2). Classification follows the general principle outlined in the first row. However, we specifically outlined the different mechanisms through which an alien species can cause impacts in order to help assessors to look at the different aspects and to identify potential research gaps. Numbers next to different impact classes reference the numbering of impacts in the classification of impact mechanisms in the GISD (Figure 1).

Second, Kumschick and colleagues [25] described scenarios of deleterious and beneficial environmental impacts by alien taxa, but here we consider only the deleterious impacts (see point 2 above). Third, we combined the two lowest ranking scenarios for each mechanism. The zero-ranked scenario in each case always refers to “No impact known or detectable,” but as the presence of an alien individual in a new environment always produces a change to the properties of an ecosystem (e.g., by altering its genetic or species diversity), by definition it has a non-zero impact in some context. Note that there is a crucial distinction between species with no known impacts, and species for which there is insufficient evidence to assess impact (see section in Box 2 on “Data Deficient” species). Finally, we edited the scenarios of Kumschick and colleagues [25] to resolve some terminological ambiguities in respect of our classifications, and to ensure that the scenarios are aligned with the mechanisms of impact identified in the GISD.

**Box 2. Description of the Categories in the Impact Classification Scheme**

The relationship between categories is shown in Figure 2. A species is considered to have a given level of impact (**MA**, **MR**, **MO**, **MI**, or **ML**) when the best available evidence indicates that it has previously had impacts in a region to which it is not native that meet any of the relevant criteria presented in Table 1. Species are categorised by the most severe impact recorded under any impact mechanism (Table 1), as follows:

**Massive (MA)**

A species is considered to have **Massive** impacts when it *leads to the replacement and local extinction of native species, and produces irreversible changes in the structure of communities and the abiotic or biotic composition of ecosystems*. Note that “local” refers to the typical spatial extent over which the original native communities can be characterised.

**Major (MR)**

A species is considered to have **Major** impacts when it *causes the local or population extinction of at least one native species, and leads to reversible changes in the structure of communities and the abiotic or biotic composition of ecosystems*, and has no impacts that cause it to be classified in the **MA** impact category.

**Moderate (MO)**

A species is considered to have **Moderate** impacts when it *causes declines in the population densities of native species, but no changes to the structure of communities or to the abiotic or biotic composition of ecosystems*, and has no impacts that would cause it to be classified in a higher impact category.

**Minor (MI)**

A species is considered to have **Minor** impacts when it *causes reductions in the fitness of individuals in the native biota, but no declines in native population densities*, and has no impacts that would cause it to be classified in a higher impact category.

**Minimal (ML)**

A species is considered to have **Minimal** impacts when it is *unlikely to have caused deleterious impacts on the native biota or abiotic environment*. Species that have been evaluated under the categorisation process but for which impacts have not been assessed in any study should not be classified in this category, but rather should be categorised as **Data Deficient**.

**Data Deficient (DD)**

A species is categorised as **Data Deficient** when the best available evidence indicates that it has individuals existing in a wild state in a region beyond the boundary of its native geographic range, but either there is inadequate information to classify the species with respect to its impact, or insufficient time has elapsed since introduction for impacts to have become apparent. It is expected that all introduced species will have an impact at some level, because by definition an alien individual in a new environment has a non-zero impact. However, listing a species as **Data Deficient** recognises that current information is insufficient to assess that level of impact.

**No Alien Populations (NA)**

A species is categorised as **No Alien Populations** when there is no reliable evidence that it has or had individuals existing in a wild state in a region beyond the boundary of its native geographic range. We assume that absence of evidence is evidence of absence in this case, as it is impossible to prove that a species has no alien individuals anywhere in the world. Species with individuals kept in captivity or cultivation in an area to which it is not native [60] would be classified here. A species could currently have no individuals existing in a wild state in a region beyond the boundary of its native geographic range because it has died out in, or has been eradicated from, such an area. In these cases, there should be evidence relating to impact that causes it to be classified in one of the impact categories (**ML**, **MI**, **MO**, **MR**, **MA**), or alternatively no evidence of impact, which would cause it to be classified as **Data Deficient**.

**Not Evaluated (NE)**

A species is **Not Evaluated** when it has not yet been evaluated against the criteria, as is also the case in the IUCN Red List [23].

**Cryptogenic (CG)**

**Cryptogenic** is not a category within the scheme presented in Figure 2, but rather a label to be applied to those taxa for which it is unclear, following evaluation, whether the individuals present at a location are native or alien [61]. This is a particular problem in the marine realm, for cosmopolitan plants and for many stored product arthropod pests, for which the native geographic ranges are unknown. Cryptogenic taxa may have deleterious impacts where they occur [62],[63]. We suggest on the basis of the precautionary principle that cryptogenic species are evaluated as if they were aliens, but that their impact categorisation is modified by the **CG** label (e.g., for a cryptogenic species with **Major** impact: *Genus species* **MR** **[CG]**).

Instead of using the impact mechanisms and scenarios to produce an overall numerical impact score for a species, we use the scenarios to assign a species to one of five sequential categories of impact: in ascending order of impact, these categories are Minimal (ML), Minor (MI), Moderate (MO), Major (MR), and Massive (MA) (Figure 2; Box 2). The process of categorisation would involve collation of all available evidence on impact for the members of a taxon from all regions to which the taxa have been introduced (or from the focal region where relevant), and using that evidence to inform expert opinion on the category of impact indicated. The impact category to which a species is assigned is that corresponding to the highest level of deleterious impact identified from any of the impact mechanisms (Box 2; Table 1). Listing of a species in a higher category explicitly assumes that there is evidence that the species has had a greater deleterious impact on some aspect of an environment in which it is alien than a species in a lower category of impact. Impact rating should be considered in the absence of management, but our approach may contribute to a process of prioritising species for management (e.g., if a new incursion by a high impact species is detected), as is required by Aichi target 9 of the Convention on Biological Diversity's Strategic Plan 2020 (www.cbd.int/sp/targets/rationale/target-9). We would expect some species to move between categories in successive categorisation processes, at the most trivial level from Not Evaluated (NE) into one of the evaluated categories (Figure 2), but subsequently from No Alien Population (NA) to an alien category (Data Deficient [DD], or one of Minimal (ML), Minor (MI), Moderate (MO), Major (MR), or Massive (MA) if introduced into the wild beyond its natural range limits), and potentially then between different categories of alien impact. Species whose alien status is uncertain can be identified as cryptogenic (CG) within any of the impact categories (Box 2).

## Uncertainty

There are likely to be many cases where uncertainty exists about the correct categorisation of a species in terms of the magnitude of its impacts, even for species for which data is considered adequate (Box 2; Table 1). Consequently, it will be sensible to include an estimate of the degree of uncertainty attached to all categorisations, so that the degree of confidence in every classification is explicitly made clear. Only epistemic or reducible uncertainty (i.e., uncertainty due to data quality) is of importance for the proposed classification. Uncertainty related to variation in impacts in space or time (stochasticity or irreducible uncertainty) is not considered because only the highest impact reported is considered. We acknowledge that there are different ways to characterise uncertainty, but we suggest for practical purposes a categorisation of uncertainty into three levels—high, medium, and low confidence—based on approaches used by the Intergovernmental Panel on Climate Change (IPCC) [27] and European and Mediterranean Plant Protection Organization (EPPO) [28],[29]. Further details are given in Tables S1 and S2 and Text S1.

## Discussion

What follows is a condensed version of our Discussion for the general reader: we encourage those with a more specific interest in the subject to read the full version, available as Text S2.

There are abundant examples of alien species having deleterious environmental impacts that alter the structure, function, or dynamics of the ecosystem concerned. The need to prioritise management responses to these impacts (or the objectively quantified risk of such threats) provides a strong impetus to develop a standardised system by which impacts can be rigorously quantified and compared in terms of their magnitudes. However, there is no commonly employed method of quantifying and ranking impacts on biodiversity and ecosystems [30]. Regulatory bodies have attempted to develop a variety of different schemes [31]–[33], but a unified standard classification does not exist. Indeed, the lack of a standard metric, coupled with data deficiencies, is likely a major reason why risk assessments rarely include quantitative evaluations of impact [34]. We believe that our proposed classification scheme (Figure 2; Table 1; Box 2) provides a pragmatic solution to some of these needs. It also has the attractive quality that it follows a similar approach to the already widely adopted Red Listing approach to categorising extinction risk, and so could theoretically be quickly integrated with existing practices and policies across the globe. It aligns with mechanisms of impact identified in the IUCN GISD (Figure 1), and hence can be used in conjunction with that important database. The interlink between the IUCN GISD and Red List may also permit a more structured application of the present scheme to the evaluation of the impact of alien species on species assessed in the Red List.

Our scheme overcomes the problems that arise from the fact that there is no standard metric of impact, or method of quantifying it. By relating quantitative studies to a set of standardised semi-quantitative scenarios enhanced by descriptions, we can identify and rank mechanisms of impact indicated by the evidence provided. Although there is often a significant degree of uncertainty surrounding the impact of any given alien species, both because of measurement error and subsequent translation of what a quantitative trait measure means in terms of actual environmental change, the broad separation of our categories in terms of the level of impact they represent means that impacts can be classified with a good degree of confidence [24]. Furthermore, our scheme includes a mechanism for appending estimates of uncertainty to each categorisation (Text S1). Similar issues of uncertainty pertain to the IUCN Red List criteria and categories (albeit that they are often overlooked), but while the precise categorisation of some species is the subject of considerable debate [35], there is little doubt that the Red List functions as an effective and credible guide to the threat of extinction and as a valuable trend indicator over time [36]. We hope that our categorisation scheme will come to be viewed in the same light.

In contrast to the previous use of such scenarios to estimate overall impact [24],[26],[37], here they are simply used to identify the mechanism by which a species has its highest impact. A lack of data on some mechanisms can affect estimates of overall impact, but does not prevent the classification of a species under our scheme, if information is available on other mechanisms of impact. Our categorisation scheme is therefore effective with less available data than required to assess the overall impact of a species.

A lack of information on some mechanisms of impact may lead to a species being placed in a lower impact category than might otherwise be the case. However, in many cases, it will be difficult to distinguish whether an alien is the driver of environmental changes, or simply a “passenger” responding to the same driver as the natives [38]. Synergistic interactions between alien species and other stressors are also possible—and perhaps increasingly common—but difficult to anticipate [39]. This suggests that categorisation will be cautious: an alien is likely to be assigned to a high impact category if it is associated with significant change, even if it is not the main driver. This is a sensible situation under the precautionary principle, where benefit of the doubt should not be given to the alien. However, our system is intended to be dynamic, allowing for updates as new or more reliable data become available, and as the documented impact history of a species unfolds through space and time [40]–[42]. In fact, the classification scheme could in practice serve to identify knowledge gaps for invaders for which there is currently little or no information.

The use of standardised scenarios allows analysis of a wide range of factors relating to impact, such as correlates of magnitude, variation, and temporal and spatial change. The category of impact to which an alien species is assigned can increase or decrease as more deleterious impacts are discovered, if the alien species is subsequently identified as a passenger rather than a driver of change, or if environmental influences change. The protocol can also be applied with minor modification to impacts at a range of spatial scales, allowing national, regional, and global categorisation of impacts. It complements and can inform national assessment schemes in which species are assigned to different lists [43]–[45] depending on whether they are species with a low risk of impact (“white list,” ML, or perhaps MI in this scheme), of assumed or uncertain impact (“grey list”), or have measurable impacts of concern (“black list,” corresponding to MO, MR, or MA) on environments. In all of these respects, the scheme is analogous to the IUCN Red List [46]. Another similarity with the IUCN Red List approach is that some impact listings, as with some threat listings, are likely to be context dependent. For example, a relatively widespread taxon may be classified as at high risk of extinction in some national Red Lists if the species is locally rare or threatened (e.g., the country is near the range edge). Similarly, an alien impact that is observed in one area of the introduced range may not occur elsewhere, or may not be as important elsewhere: invasiveness, and by extension impact, is a characteristic of a population rather than a species [2],[47]. Overall, the assessment of impacts at more restricted scales may predominantly depend on evidence of impacts elsewhere (which may be subject to higher error, given context-dependent variation), whereas at large scales, information on impacts will increasingly derive from the focal region.

All of this highlights the importance of ensuring that the impacts of aliens on populations and communities are measured at an appropriate spatial scale, taking into account the typical spatial size at which original native communities can be characterised (termed the “local scale” here). Studies at very restricted spatial scales (i.e., patches of 10s or 100s of square metres) might overestimate impacts if extrapolated to larger scales, while studies at extensive spatial scales (i.e., regional or national) might underestimate them. For example, an alien species might be shown in a field experiment to exclude natives from areas the size of experimental plots, and perhaps even to extirpate natives from entire habitat patches, without having a significant effect on community diversity (e.g., because of the influence of spatial dynamics, refugia, or rescue effects). In this case, it is likely that populations of some natives would have declined (e.g., competitors or food species) in the habitats in which the alien species occurs, without resulting in local extinctions: the appropriate classification under our scheme would therefore be MO in this case (Table 1). This approach has the benefit of identifying impacts demonstrated in very small habitat patches that may be a cause for greater concern in the future.

One shortcoming of the proposed classification scheme is that it is not designed to be predictive by itself. For example, it cannot be applied to species with no previous history of alien populations (if evaluated, these species cannot be classified other than NA), and, as recorded impacts usually accrue with population growth, species that have not been introduced for long (short residence times; Box 1) or not introduced in large numbers (low propagule pressures; Box 1) are likely to receive a low rating. Nevertheless, the scheme could provide predictive information on the likely magnitude of impacts of a species, if it is phylogenetically or functionally similar to a species that has known impacts as an alien on the native biota or abiotic environment [33], or if there is a mechanistic understanding of how impacts might progress. This may be helpful given that a history of impact elsewhere is currently often considered to be the best available predictor of the impact potential of an alien species [40],[48],[49], but is of no use for predicting impacts of species with no alien populations. Such species could be assessed under our scheme, but with their categorisation assigned a high level of uncertainty. We do not advocate that such approaches substitute for the precautionary principle in cases of species with unknown impacts, but they may nevertheless help to understand which species may be most damaging if introduced. A future development of the scheme would be to include an estimate of potential impact for such species.

## Supporting Information

Figure S1 **The relationship between the overall potential environmental impact score and the impact category to which the species is assigned under our classification scheme, for data on alien mammals in Europe (from [9]** **).** Environmental impact score is the sum of the impacts over the six categories given by Kumschick and colleagues (39). Species are assigned to impact category on the basis of the largest impact value in any of the six categories. Note that Kumschick and colleagues (39) do not score impacts under several of the classes listed in Table 1. The analysis is confined to impacts recorded for species in their alien ranges in Europe (indicating the scalable nature of our approach): a global analysis might shift some species to higher impact categories. Note that the data points have been jittered to improve visibility. Impact score and category are clearly positively related, but some species can have higher scores than other species in higher categories.
(TIF)

Figure S2 **Relationship between the overall environmental impact of European alien plants (the median score across all assessed classes of impact.** **Note that not all classes of impact in Table 1 were assessed) and the impact classification assigned under our scheme (defined by the highest score achieved in any of the impact classes).** Species with names indicated have, compared to their average impact across the classes assessed, a disproportionally strong impact in one individual class. While their high impact may be overlooked when assessing the overall impact, it is captured by our suggested classification scheme under which species are assigned on the basis of maximum, not average, impact. For example, *Cortaderia selloana* exerts a strong impact (**MA**) on ecosystem processes, its impacts in other classes being **MO** at most. Note that data points have been jittered to improve visibility. Based on M. Vilà, Z. Marková, P. Pyšek, J. Pergl (unpublished data) following the impact assessment methodology of [10].
(TIF)

Table S1 **Guidance regarding the use of the confidence rating (modified from the EPPO pest risk assessment decision support scheme [2],[64])**.
(DOCX)

Table S2 **Suggested distribution of likelihoods (in percent) of the impact of alien species being in a certain category depending on the confidence of the assessment.** Probability distributions follow a standardised beta distribution with parameters α and β. The histogram below the table provides a pictorial representation of the same probabilities.
(DOCX)

Text S1 **Categorising uncertainty.**
(DOCX)

Text S2 **Full version of the** **Discussion** **.**
(DOCX)

## Abbreviations

CG: cryptogenic
DD: Data Deficient
GISD: Global Invasive Species Database
IUCN: International Union for Conservation of Nature
MA: Massive
MI: Minor
ML: Minimal
MO: Moderate
MR: Major
NA: No Alien Population
NE: Not Evaluated
SSC: Species Survival Commission
