# Supplementary figures and images for: A Unified Classification of Alien Species Based on the Magnitude of their Environmental Impacts
Source: PLoS Biol. 2014 May 6;12(5):e1001850. doi: 10.1371/journal.pbio.1001850 (PMC4011680; doi:10.1371/journal.pbio.1001850)

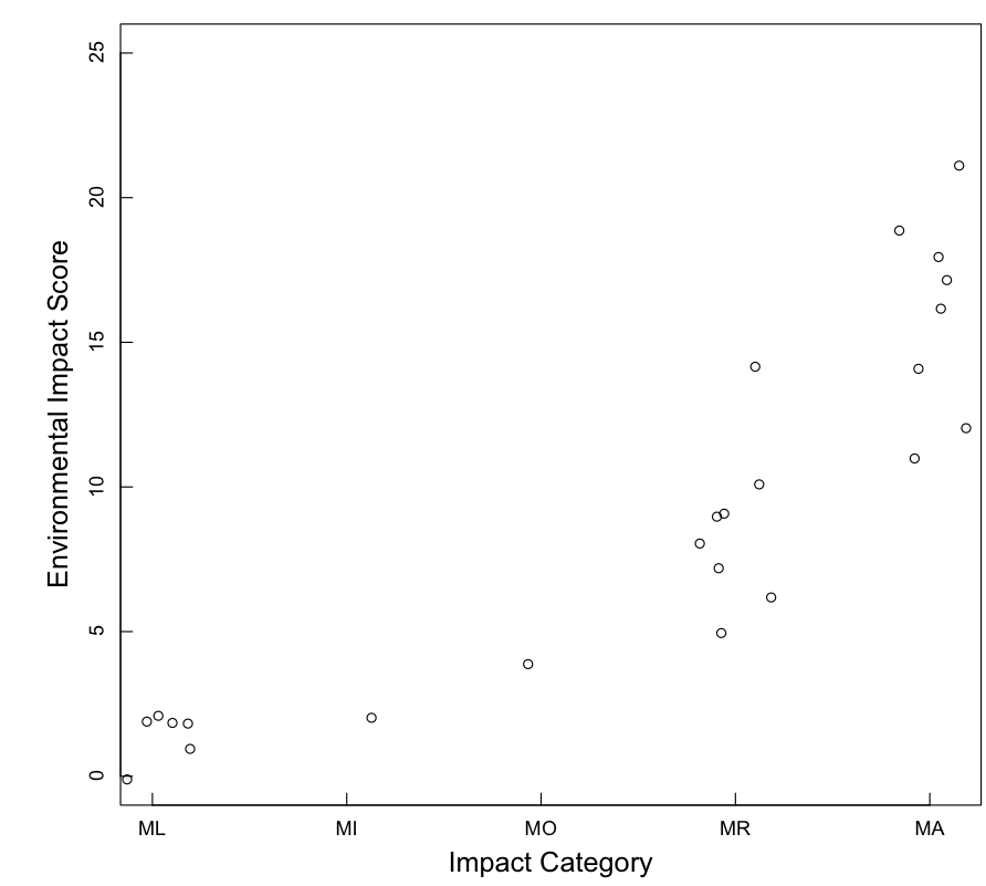

Supplement: Figure S1 — The relationship between the overall potential environmental impact score and the impact category to which the species is assigned under our classification scheme, for data on alien mammals in Europe (from [9] ). Environmental impact score is the sum of the impacts over the six categories given by Kumschick and colleagues (39). Species are assigned to impact category on the basis of the largest impact value in any of the six categories. Note that Kumschick and colleagues (39) do not score impacts under several of the classes listed in Table 1. The analysis is confined to impacts recorded for species in their alien ranges in Europe (indicating the scalable nature of our approach): a global analysis might shift some species to higher impact categories. Note that the data points have been jittered to improve visibility. Impact score and category are clearly positively related, but some species can have higher scores than other species in higher categories. (TIF) [file pbio.1001850.s001.tif]

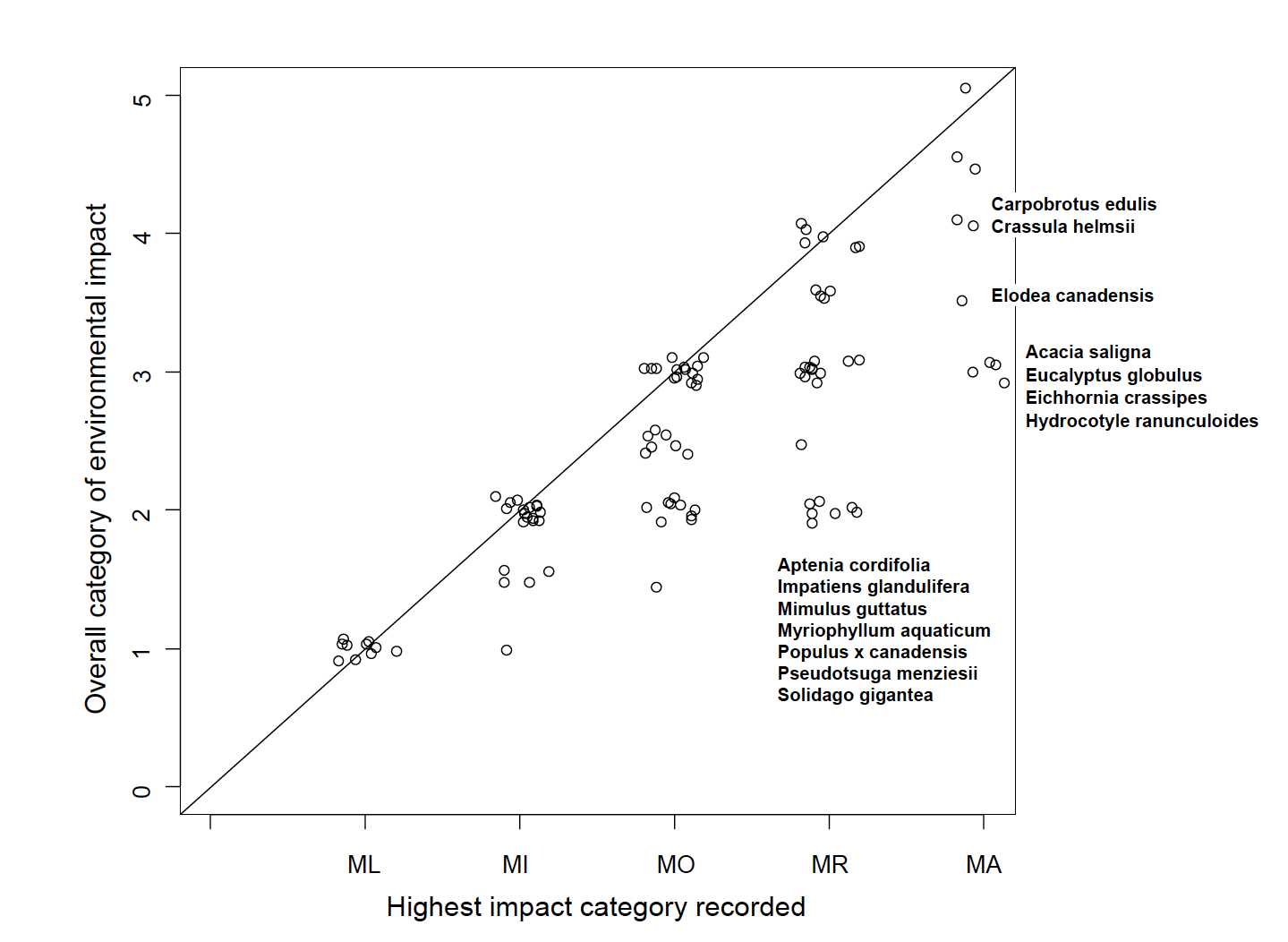

Supplement: Figure S2 — Relationship between the overall environmental impact of European alien plants (the median score across all assessed classes of impact. Note that not all classes of impact in Table 1 were assessed) and the impact classification assigned under our scheme (defined by the highest score achieved in any of the impact classes). Species with names indicated have, compared to their average impact across the classes assessed, a disproportionally strong impact in one individual class. While their high impact may be overlooked when assessing the overall impact, it is captured by our suggested classification scheme under which species are assigned on the basis of maximum, not average, impact. For example, Cortaderia selloana exerts a strong impact (MA) on ecosystem processes, its impacts in other classes being MO at most. Note that data points have been jittered to improve visibility. Based on M. Vilà, Z. Marková, P. Pyšek, J. Pergl (unpublished data) following the impact assessment methodology of [10]. (TIF) [file pbio.1001850.s002.tif]
