# Supplementary material for: A Unified Classification of Alien Species Based on the Magnitude of their Environmental Impacts
Source: PLoS Biol. 2014 May 6;12(5):e1001850. doi: 10.1371/journal.pbio.1001850 (PMC4011680; doi:10.1371/journal.pbio.1001850)
Supplement: Table S1 — Guidance regarding the use of the confidence rating (modified from the EPPO pest risk assessment decision support scheme [2],[64]). (DOCX) [file pbio.1001850.s003.docx]

**Table S1**. Guidance regarding the use of the confidence rating (modified from the EPPO pest risk assessment decision support scheme (Alan MacLeod 09/03/2011; revised 28/04/2011; copied from CAPRA, version 2.74; *2*)).

| Confidence level | Examples |
| --- | --- |
| High | There is direct relevant evidence to support the assessment.  The situation can easily be predicted. There are reliable/good quality data sources on impacts of the species. The interpretation of data/information is straightforward. Data/information are not controversial, contradictory. |
| Medium | There is some evidence to support the assessment. Some information is indirect, e.g. data from phylogenetically or functionally similar species have been used as supporting evidence.  The interpretation of the data is to some extent ambiguous or contradictory. |
| Low | There is no direct evidence to support the assessment, e.g. only data from other species have been used as supporting evidence. Evidence is poor and difficult to interpret, e.g. because it is strongly ambiguous. The information sources are considered to be of low quality or contain information that is unreliable. |
