# Supplementary material for: A Unified Classification of Alien Species Based on the Magnitude of their Environmental Impacts
Source: PLoS Biol. 2014 May 6;12(5):e1001850. doi: 10.1371/journal.pbio.1001850 (PMC4011680; doi:10.1371/journal.pbio.1001850)

**Table S2**. Suggested distribution of likelihoods (in percent) of the impact of alien species being in a certain category depending on the confidence of the assessment. Probability distributions follow a standardized beta distribution with parameters α and β. The histogram below the table provides a pictorial representation of the same probabilities.


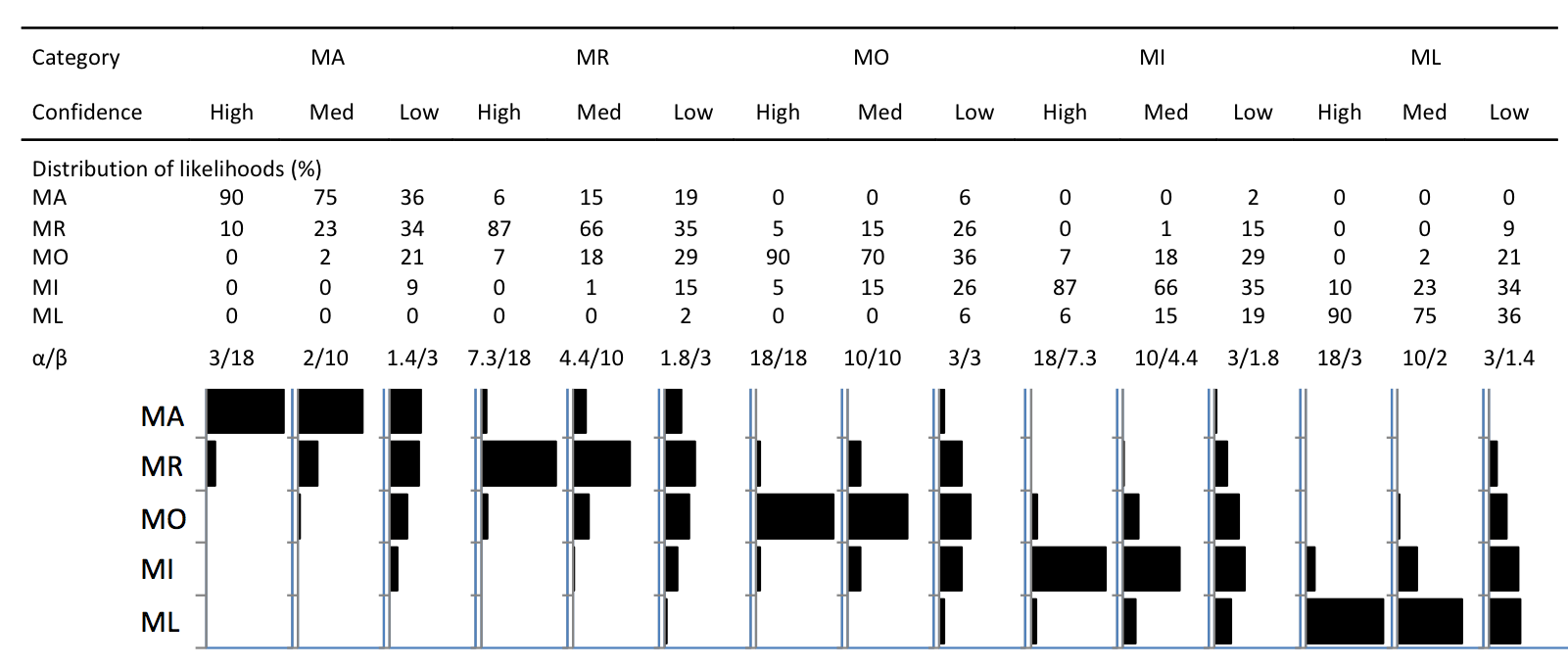

Supplement: Table S2 — Suggested distribution of likelihoods (in percent) of the impact of alien species being in a certain category depending on the confidence of the assessment. Probability distributions follow a standardised beta distribution with parameters α and β. The histogram below the table provides a pictorial representation of the same probabilities. (DOCX) [file pbio.1001850.s004.docx]
