# Supplementary material for: A Unified Classification of Alien Species Based on the Magnitude of their Environmental Impacts
Source: PLoS Biol. 2014 May 6;12(5):e1001850. doi: 10.1371/journal.pbio.1001850 (PMC4011680; doi:10.1371/journal.pbio.1001850)

**Categorising Uncertainty**

For each species that is assessed and for which adequate data exist to allow for categorization, the assessor places it in the most likely of the five categories (**MA**, **MR**, **MO**, **MI**, **ML**) and assigns a level of confidence to this placement according to the availability and reliability of evidence, and whether or not the evidence is contradictory. *High confidence* is assigned when there is direct and relevant evidence to support the assessment, the data are reliable and of good quality, and all evidence points in the same direction. *Medium confidence* is assigned when there is some evidence to support the assessment, but some of the data are indirect (estimated from another phylogenetically or functionally similar alien species with recorded impact, or deriving from a probabilistic risk assessment) and/or there is some degree of ambiguity in the direction or magnitude of the impact. *Low confidence* is defined as no direct evidence to support the assessment, for example only data from other species have been used as supporting evidence or data are of low quality or strongly ambiguous. More detailed descriptions of confidence levels are given in Table S1. Confidence levels are translated into arbitrary probabilities that the assigned category is the correct one. *High confidence* means that the assessor feels they have approximately 90% chance of the given score being correct. *Medium confidence* was defined as 65-75% chance of the assessor score being correct and *Low confidence* only 35% chance of being correct.

These assessments of confidence mean that there is some probability that a species should in reality be assigned to another category (most likely to a neighbour in Fig. 2 in the main document). This probability will be lowest for species categorized with *High confidence,* and highest for species categorized with *Low confidence*. We estimated the distribution of the remaining probability in each case by assigning it amongst the other categories according to a beta distribution with parameters chosen such that the assigned category had the highest probability and the variance in confidence increased from *High* to *Medium* to *Low*, taking approximate values of 0.007, 0.011, and 0.038, respectively (Table S2). The suggested distributions of likelihoods together with the descriptions of uncertainties in Table S1 should serve as guidance for assessors to assign confidence levels to their assessments. A choice of predefined distributions offers a consistent way to infer a rating distribution from a single confidence rating, but we suggest that assessors examine these distributions carefully to make sure they accord with their own perception of confidence (*1*).

*Supplemental Reference*

1. Holt J, Leach, AW, Knight JD, Griessinger D, MacLeod,A, et al. (2012) Tools for visualizing and integrating pest risk assessment ratings and uncertainties. EPPO Bull 42: 35-41.

2. Griessinger D, Suffert M, Brunel S, Petter F (2012) CAPRA: the EPPO Computer Assisted PRA scheme. EPPO Bull 42: 42-47.

**Table S1**. Guidance regarding the use of the confidence rating (modified from the EPPO pest risk assessment decision support scheme (Alan MacLeod 09/03/2011; revised 28/04/2011; copied from CAPRA, version 2.74; *2*)).

| Confidence level | Examples |
| --- | --- |
| High | There is direct relevant evidence to support the assessment.  The situation can easily be predicted. There are reliable/good quality data sources on impacts of the species. The interpretation of data/information is straightforward. Data/information are not controversial, contradictory. |
| Medium | There is some evidence to support the assessment. Some information is indirect, e.g. data from phylogenetically or functionally similar species have been used as supporting evidence.  The interpretation of the data is to some extent ambiguous or contradictory. |
| Low | There is no direct evidence to support the assessment, e.g. only data from other species have been used as supporting evidence. Evidence is poor and difficult to interpret, e.g. because it is strongly ambiguous. The information sources are considered to be of low quality or contain information that is unreliable. |

**Table S2**. Suggested distribution of likelihoods (in percent) of the impact of alien species being in a certain category depending on the confidence of the assessment. Probability distributions follow a standardized beta distribution with parameters α and β. The histogram below the table provides a pictorial representation of the same probabilities.


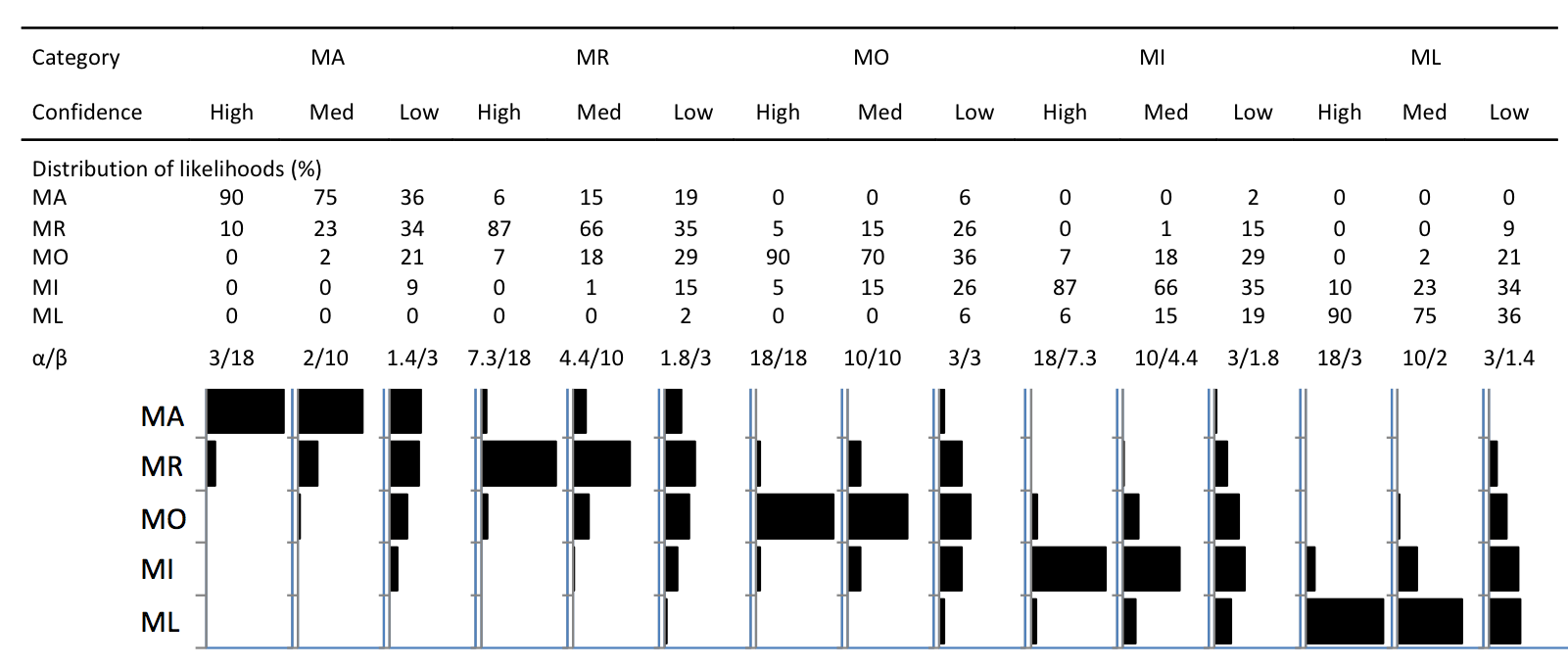

Supplement: Text S1 — Categorising uncertainty. (DOCX) [file pbio.1001850.s005.docx]
