# Supplementary material for: A Unified Classification of Alien Species Based on the Magnitude of their Environmental Impacts
Source: PLoS Biol. 2014 May 6;12(5):e1001850. doi: 10.1371/journal.pbio.1001850 (PMC4011680; doi:10.1371/journal.pbio.1001850)
Supplement: Text S2 — Full version of the Discussion . (DOCX) [file pbio.1001850.s006.docx]

**Text S2**

**Full Version of the Discussion**

There are abundant examples of alien species having deleterious environmental impacts that alter the structure, function or dynamics of the ecosystem concerned. The need to prioritise management responses to these impacts (or the objectively quantified risk of such threats) provides a strong impetus to develop a standardized system by which impacts can be rigorously quantified and compared in terms of their magnitudes. However, there is no commonly employed method of quantifying and ranking impacts on biodiversity and ecosystems [*1*]. Regulatory bodies have attempted to develop a variety of different schemes [*2 – 4*] but a unified standard classification does not exist. Indeed, the lack of a standard metric, coupled with data deficiencies, is likely a major reason why risk assessments rarely include quantitative evaluations of impact [*5*]. We believe that our proposed classification scheme provides a pragmatic solution to some of these needs. It also has the attractive quality that it follows a similar approach to the already widely adopted Red Listing approach to categorizing extinction risk, and so could theoretically be quickly integrated with existing practices and policies across the globe. It aligns with mechanisms of impact identified in the IUCN GISD (Fig. 1), and hence can be used in conjunction with that important database. The interlink between the IUCN GISD and Red List may also permit a more structured application of the present scheme to the evaluation of the impact of alien species on species assessed in the Red List.

Our scheme overcomes the problems that arise from the fact that there is no standard metric of impact, or method of quantifying it. By relating quantitative studies to a set of standardized semi-quantitative scenarios enhanced by descriptions, we can identify and rank mechanisms of impact indicated by the evidence provided. Although there is often a significant degree of uncertainty surrounding the impact of any given alien species, both because of measurement error and subsequent translation of what a quantitative trait measure means in terms of actual environmental change, the broad separation of our categories in terms of the level of impact they represent means that impacts can be classified with a good degree of confidence [*6*]. Furthermore, our scheme includes a mechanism for appending estimates of uncertainty to each categorization (Text S1). Similar issues of uncertainty pertain to the IUCN Red List criteria and categories (albeit that they are often overlooked), but while the precise categorization of some species is the subject of considerable debate [*7*], there is little doubt that the Red List functions as an effective and credible guide to the threat of extinction and as a valuable trend indicator over time [*8*]. We hope that our categorization scheme will come to be viewed in the same light.

In contrast to the previous use of such scenarios to estimate overall impact [*6, 9, 10*], here they are simply used to identify the mechanism by which a species has its highest impact. For example, alien populations of the brown tree snake may have had little in the way of impact via hybridization or the spread of disease to native species, but they have clearly had critical impacts through predation on naïve island vertebrate populations [*11*] and plant reproduction [*12*], thus irreversibly changing local communities. For this reason, further introductions of the snake, especially to oceanic islands, would be contraindicated by their classification in the highest impact category in our scheme (**MA**). Moreover, lack of data on some mechanisms of impact does not prevent the classification of a species, if information is available on other mechanisms of impact. For example, information on whether the brown tree snake has impacted the chemical, physical or structural environment may be lacking, but this does not affect its categorization as **MA**. Our categorization scheme is therefore effective with less available data than required to assess the overall impact of a species, although clearly a lack of information on some mechanisms of impact may lead to a species being placed in a different impact category than might otherwise be the case. Conversely, a species for which abundant data are available, and which scores highly under several impact mechanisms, will receive the same categorization as a species that scores highly in only one impact mechanism. This is a logical consequence of categorizing species on the basis of the worst-case scenario. We see no reason for summing impacts across impact mechanisms, where data are available: for example, two **MO** impacts (i.e. changes in populations, but not in communities, through two different mechanisms) do not equate to one **MR** or **MA** impact, where a single mechanism leads to changes at the community level. Thus, our classification is guided by the level of change (individual – population – community) regardless of the type and number of mechanism(s) involved. However, the certainty with which a species is categorized is likely to increase with data availability. In practice, we suspect that species with high overall environmental impacts will also tend to be classified in higher impact categories under our scheme; preliminary analyses of environmental impacts of alien mammal (Fig. S1) and plant (Fig. S2) species in Europe are consistent with this suspicion, while also identifying outlying species with disproportionally strong impacts from one individual impact mechanism.

One shortcoming of the proposed classification scheme is that it is not designed to be predictive by itself. For example, it cannot be applied to species with no previous history of alien populations (if evaluated, these species cannot be classified other than **NA**), and, as impacts usually accrue with population growth, species that have not been introduced for long or in large numbers are likely to receive a low rating. Nevertheless, the scheme could provide predictive information on the likely magnitude of impacts of a species, if it is phylogenetically or functionally similar to a species that has known impacts as an alien on the native biota or abiotic environment [*13*], or if there is a mechanistic understanding of how impacts might progress. This may be helpful given that a history of impact elsewhere is currently often considered to be the best available predictor of the impact potential of an alien species [*13-15*], but is of no use for predicting impacts of species with no alien populations. Such species could be assessed under our scheme, but with their categorization assigned a high level of uncertainty. This is most likely to be relevant for identifying species related to aliens with known high levels of impact (**MO**, **MR** or **MA**), which may have potential for high levels of impact themselves. Information on the known impacts of alien species could also be used to model impacts in relation to trait data [*9*], to produce better predictions of the likelihood of impact magnitude for species without alien populations. Ideally, such analyses would account for residence time [*16*] to ensure that differences in impact are not a consequence of longer time periods for impacts to have developed in some taxa. Analogous studies have used the IUCN Red List to test for correlates of extinction risk [*17 – 19*]. We do not advocate that such approaches substitute for the precautionary principle in cases of species with unknown impacts, but they may nevertheless help to understand which species may be most damaging if introduced. A future development of the scheme would be to include an estimate of potential impact for such species.

Clearly, the proposed classification scheme is only as good as the evidence available on species impacts [*20*], as is true of any scheme. It is likely that much of the evidence on impacts will be observational: for example, comparison of the state or function of habitats with or without the alien. Observational evidence is relatively easy to obtain, but provides only weak inference [*21, 22*]. In such cases, it will therefore be difficult to distinguish whether the alien is the driver of any changes, or simply a “passenger” responding to the same driver as the natives [*23*]; synergistic interactions between alien species and other stressors are also possible – perhaps increasingly common – but difficult to anticipate [*24*]. This suggests that categorization will be cautious: an alien is likely to be assigned to a high impact category if it is associated with significant change, even if it is not the main driver. This is a sensible situation under the precautionary principle, where benefit of the doubt should not be given to the alien. However, our system is intended to be dynamic, allowing for updates as new or more reliable data become available, and as the documented impact history of a species unfolds through space and time [*13, 25, 26*]. In fact, the classification scheme could in practice serve to identify knowledge gaps for invaders for which there is currently little or no information. The scheme also allows an explicit statement about the (un)certainty associated with any categorization (Text S1), which may also help in the prioritization of future studies.

We have explicitly limited categorization under the proposed scheme to deleterious impacts of species – i.e. those that change the environment in such a way as to reduce native biodiversity or alter ecosystem function to the detriment of the incumbent native species (see point 2 in the Introduction). Our view is that it is important to document the negative consequences of all introductions in order to inform management decisions. Nevertheless, our categorization scheme could be a good starting point for identifying alien species for which deleterious impacts on native ecosystems are low, for example for consideration as future pets or garden ornamentals [*27*]. Any such uses should be mindful of impact history, however, and especially the fact that some alien species may currently be classified in low impact categories simply because of shorter residence times or sparser information.

The use of standardized scenarios allows analysis of a wide range of factors relating to impact, such as correlates of magnitude, variation and temporal and spatial change. The category of impact to which an alien species is assigned can increase or decrease as more deleterious impacts are discovered, if the alien species is subsequently identified as a passenger rather than a driver of change, or if environmental influences change. The protocol can also be applied with minor modification to impacts at a range of spatial scales, allowing national, regional, and global categorization of impacts. It complements and can inform national assessment schemes in which species are assigned to different lists [*28 – 30*] depending on whether they are species with a low risk of impact (“white list”, **ML** or perhaps **MI** in this scheme), of assumed or uncertain impact (“grey list”), or have measurable impacts of concern (“black list”, corresponding to **MO, MR** or **MA**) on native biodiversity. In all of these respects, the scheme is analogous to the IUCN Red List categories [*31*].

Another similarity with the IUCN Red List approach is that some impact listings, as with some threat listings, are likely to be context dependent. For example, a relatively widespread taxon may be classified as at high risk of extinction in some National Red Lists if the species is locally rare or threatened (e.g. the country is near the range edge). Similarly, an alien impact that is observed in one area of the introduced range may not occur elsewhere, or may not be as important elsewhere: invasiveness, and by extension impact, is a characteristic of a population rather than a species [*32, 33*]. For example, hybridization risk is as much a function of the resident community as it is of the alien species. Thus, *Hyacinthoides hispanica* is an alien plant that is a hybridization risk with native bluebells in the UK [*34*], but not in New Zealand where it is naturalized but where there are no native congeners for hybridization to be a problem. However, these context dependencies have different consequences for the listings of native threat versus alien species impact. National-scale extinction threat does not necessarily translate into global scale extinction threat under the IUCN Red List, whereas national-scale impact would translate into global scale impact under our categorization scheme. This might be viewed as a shortcoming of our scheme but reflects the precautionary principle for alien impacts. It is important to apply this principle given that most species that are alien and have impacts somewhere have not been introduced to many of the locations where they could potentially thrive (and have impacts). Indeed, there is evidence that many alien species can have strong impacts in at least part of their invaded range, if distributed sufficiently widely [*35, 36*]. As such, management and policy decisions should ideally be informed by a mechanistic understanding of how the impacts arise [*32*]. For example, the invasion of trees to treeless ecosystems will almost certainly be **MR** or **MA** as species over-top native vegetation, but if the same species are introduced to forested areas the impacts might be **MI** or **MO** [*37, 38*]. However, unless the context and mechanisms are well known and understood, species observed to have **MA** or **MR** impacts in any part of their introduced range should be treated with extreme caution. Overall, the assessment of impacts at more restricted scales may predominantly depend on evidence of impacts elsewhere (which may be subject to higher error, given context-dependent variation), whereas at large scales, information on impacts will increasingly derive from the focal region.

All of this highlights the importance of ensuring that the impacts of aliens on populations and communities are measured at an appropriate spatial scale, taking into account the typical spatial size at which original native communities can be characterized (termed the ‘local scale’ here). Studies at very restricted spatial scales (i.e. patches of 10s or 100s of square metres) might overestimate impacts if extrapolated to larger scales, while studies at extensive spatial scales (i.e. regional or national) might underestimate them. For example, an alien species might be shown in a field experiment to exclude natives from areas the size of experimental plots, and perhaps even to extirpate natives from entire habitat patches, without having a significant effect on community diversity (e.g. because of the influence spatial dynamics, refugia, or rescue effects). In this case, it is likely that populations of some natives would have declined (e.g. competitors or food species) in the habitats in which the alien species occurs, without resulting in local extinctions: the appropriate classification under our scheme would therefore be **MO** in this case (Table 1). This approach has the benefit of identifying impacts demonstrated in very small habitat patches that may be a cause for greater concern in the future.

In summary, the classification scheme proposed here provides a simple but general process to categorize alien species in terms of their impacts in the environment to which they have been introduced. Like the IUCN Red List, it can be used to identify priority species for action, as required by the global policies on biological invasions, as well as to facilitate comparisons of alien species impacts across taxa, time and space, and eventually to predict trajectories into the future. Mechanisms of impact are explicitly aligned with those identified in the IUCN GISD (Fig. 1), so that this classification scheme can be used in conjunction with that database. The next step is clearly to demonstrate the utility of the approach through a case study of impacts for a group of species at the global scale. This will be the subject of future work. In the long term, we anticipate significant value in adopting our scheme as a standard, and applying it to all known alien species. Once again, this is analogous to the approach developed by the IUCN for the Red List that in the last twenty years has been applied to more than 70,000 species globally, with complete global assessments for several key taxonomic groups.

**References**

1. Parker IM, Simberloff D, Lonsdale WM, Goodell K, Wonham M, et al. (1999) Impact: Toward a framework for understanding the ecological effects of invaders. Biol Inv 1: 3-19.

2. FAO (2004) Pest risk analysis for quarantine pests including analysis of environmental risks and living modiﬁed organisms, ISPM 11. Rome: International Plant Protection Convention, FAO.

3. EPPO (2007) Guidelines on Pest Risk Analysis; Decision support scheme for quarantine pests, EPPO standard PM 5⁄3 (3).

4. EFSA Panel on Plant Health (PLH) (2011) Guidance on the environmental risk assessment of plant pests. EFSA Journal 9: 2460.

5. Leung B, Roura-Pascual N, Bacher S, Heikkilä J, Brotons L, et al. (2012) TEASIng apart alien species risk assessments: a framework for best practices. Ecol Lett 15: 1475-1493.

6. Nentwig W, Kühnel E, Bacher S (2010) A Generic Impact-Scoring System Applied to Alien Mammals in Europe. Conserv Biol 24: 302-311.

7. Davies TD, Baum JK (2012) Extinction risk and overfishing: Reconciling conservation and fisheries perspectives on the status of marine fishes. Sci Rep 2: 561.

8. Butchart SHM (2008) Red List Indices to measure the sustainability of species use and impacts of invasive alien species. Bird Conserv Int 18: S245-S262.

9. Kumschick S, Bacher S, Blackburn TM (2013) What determines the impact of alien birds and mammals in Europe? Biol Inv 15: 785-797.

10. Kumschick S, Nentwig W (2010) Some alien birds have as severe an impact as the most effectual alien mammals in Europe. Biol Conserv 143: 2757-2762.

11. Fritts TH, Rodda GH (1998) The role of introduced species in the degradation of island ecosystems: A case history of Guam. Annu Rev Ecol Syst 29: 113-140.

12. Mortensen HS, Dupont YL (2008) Snake in paradise: Disturbance of plant reproduction following extirpation of bird flower-visitors on Guam. Biol Conserv 141: 2146-2154.

13. Ricciardi A (2003) Predicting the impacts of an introduced species from its invasion history: an empirical approach applied to zebra mussel invasions. Freshw Biol 48: 972-981.

14. Grosholz ED, Ruiz GM (1996) Predicting the impact of introduced marine species: lessons from the multiple invasions of the European green crab *Carcinus maenas.* Biol Conserv 78: 59-66.

15. Williamson M (1996) Biological invasions. London: Chapman and Hall

16. Wilson JRU, Richardson DM, Rouget M, Proçhes S, Amis MA et al. (2007) Residence time and potential range: crucial considerations in modelling plant invasions. Divers Distrib 13: 11-22.

17. Gaston KJ, Blackburn TM (1995) Birds, body size and the threat of extinction. Philos Trans R Soc Lond B 347: 205-212.

18. Cardillo M (2003) Biological determinants of extinction risk: why are smaller species less vulnerable? Anim Conserv 6: 63-69.

19. Cardillo M, Mace GM, Gittleman JL, Jones KE, Bielby J, Purvis A (2008) The predictability of extinction: biological and external correlates of decline in mammals. Proc R Soc Lond B 275: 1441-1448.

20. Strubbe D, Shwartz A, Chiron F (2011) Concerns regarding the scientific evidence informing impact risk assessment and management recommendations for invasive birds. Biol Conserv 144: 2112-2118.

21. Diamond J (1986) Overview: laboratory experiments, field experiments, and natural experiments. In: Diamond J, Case TJ, editors. Community Ecology. New York: Harper Row. pp. 3- 22.

22. McArdle BH (1996) Levels of evidence in studies of competition, predation, and disease. NZ J Ecol. 20: 7-15.

23. MacDougall AS, Turkington R (2005) Are invasive species the drivers or passengers of ecological change in highly disturbed plant communities? Ecology 86: 42-55.

24. Didham RK, Tylianakis JM, Gemmell NJ, Rand TA, Ewers RM (2007) Interactive effects of habitat modification and species invasion on native species decline. Trends Ecol Evol 22: 489–496.

25. Strayer DL, Eviner VT, Jeschke JM, Pace ML (2006) Understanding the long-term effects of species invasions. Trends Ecol Evol 21: 645–651.

26. Dostál P, Müllerova J, Pyšek P, Pergl J, Klinerova T (2013) The impact of an invasive plant changes over time. Ecol Lett 16: 1277-1284.

27. Dehnen-Schmutz K (2011) Determining non-invasiveness in ornamental plants to build green lists. J Appl Ecol 48: 1374-1380.

28. Branquart E editor (2007) Guidelines for environmental impact assessment and list classification of non-native organisms in Belgium, Version 2.5. http://www.ias.biodiversity.be.

29. Essl F, Nehring S, Klingenstein F, Milasowszky N, Nowack C, et al (2011) Review of risk assessment systems of IAS in Europe and introducing the German-Austrian Black List Information System (GABLIS). J Nat Conserv 19: 339-350.

30. Sandvik H, Sæther B-E, Holmern T, Tufto, J, Engen S, Roy HE (2013) Generic ecological impact assessments of alien species in Norway: a semi-quantitative set of criteria. Biodivers Conserv 22: 37-62.

31. Mace GM, Collar NJ, Gaston KJ, Hilton-Taylor C, Akçakaya HR, et al. (2008) Quantification of extinction risk: IUCN's system for classifying threatened species. Conserv Biol 22: 1424-1442.

32. Ricciardi A, Hoopes MF, Marchetti MP, Lockwood JL (2013) Progress toward understanding the ecological impacts of non-native species. Ecol Mon 83: 263-282.

33. Simberloff D, Martin JL, Genovesi P, Maris V, Wardle DA, et al. (2013) Impact of biological invasions: what's what and the way forward. Trends Ecol Evol 28: 58–66.

34. Kohn, D, Hulme PE, Hollingsworth P, Butler A (2009) Are native bluebells (*Hyacinthoides non-scripta*) at risk from alien congenerics? Evidence from distributions and co-occurrence in Scotland. Biol Conserv 142: 61-74.

35. Rejmanek M, Randall JM (2004) The total number of naturalized species can be a reliable predictor of the total number of alien pest species. Divers Distrib 10: 367–369.

36. Ricciardi A, Kipp R (2008) Predicting the number of ecologically harmful species in an aquatic system. Divers Distrib 14: 374-380.

37. Jäger H, Tye A, Kowarik I (2007) Tree invasion in naturally treeless environments: Impacts of quinine (*Cinchona pubescens*) trees on native vegetation in Galapagos. Biol Conserv 140: 297-307.

38. Rundel PW Dickie IE, Richardson DM (2014) Tree invasions into treeless areas: mechanisms and ecosystem processes. Biol Inv 16: 663-675.
